# Antimicrobial and Oxidative Activities of Different Levels of Silver-Exchanged Zeolites X and ZSM-5 and Their Ecotoxicity

**DOI:** 10.3390/ph17121586

**Published:** 2024-11-25

**Authors:** Elitsa L. Pavlova, Elena P. Nenova, Lyubomira D. Yocheva, Iliana A. Ivanova, Peter A. Georgiev

**Affiliations:** 1 Faculty of Physics, Sofia University “St. Kliment Ohridski”, 5 James Bourchier Blvd, 1164 Sofia, Bulgaria; 2 Faculty of Biology, Sofia University “St. Kliment Ohridski”, 8 Dragan Tsankov Blvd, 1164 Sofia, Bulgaria; elena_pnenova@abv.bg (E.P.N.); ilivanova@abv.bg (I.A.I.); 3 Faculty of Medicine, Sofia University “St. Kliment Ohridski, 1 Kozyak Str, 1407 Sofia, Bulgaria; lyubomirayocheva@abv.bg

**Keywords:** Ag species, antimicrobial activity, ecotoxicity, luminescence, reactive oxygen species, zeolites

## Abstract

Objectives: The antimicrobial, oxidative activities, and ecotoxicity of synthesized silver-loaded zeolites (X and ZSM-5(MFI), Si-to-Al ratios 12 and 25) were studied, linking antimicrobial properties to material structure and released active silver species. Methods: The materials were characterized by SEM, EDX, TEM, and XRPD. All materials, with a silver content of 1–3%wt for the Ss and about 35%wt for the X-zeolites, were tested against *Escherichia coli* and *Staphylococcus aureus*. Redox activity was studied in physiological (pH 7.4/37 °C) and optimal (pH 8.5/37 °C) conditions in chemiluminescent model systems. In the ecotoxicity tests, we used *Daphnia magna.* Results: A proportional correlation was observed between the bactericidal effect of and the silver content in the zeolites. AgX with a Si/Al ratio of ~1.23 and 35% silver showed a higher antimicrobial efficiency, particularly against Gram-negative *E. coli* versus Gram-positive *S. aureus*. The concentration thresholds were as follows: AgXas had a bactericidal effect at 0.003 g/L^−1^, with an MIC at 0.0015 m/L^−1^ for *E. coli*; SA25-Ag, AgXcl, AgXrc had a bactericidal effect at 2.5 g/L^−1^. The bacteria were more resilient than *Daphnia magna*, which showed a 90–100% lethality at Ag–zeolite concentrations of 0.00625 to 0.0125 g/L^−1^. AgXas and AgXrc demonstrated strong reactive oxygen species generation at both the physiological and optimal pH, explaining their bactericidal effects. In general, the tested materials showed an inhibition of the generated reactive oxygen species depending on the model system and conditions. Conclusions: The silver species leached from the new materials explain their higher oxidation and bactericidal activity. While suitable for stringently controlled biological applications, their release into the environment, in concentrations higher than 0.01g/L^−1^, should be avoided.

## 1. Introduction

Antimicrobial resistance (AMR) has been identified as one of the top public health and societal welfare threats from a global and long-term perspective [1]. The observed persistent increase in antibiotic resistance in pathogens and the substantial environmental pollution from antibiotics request an urgent scientific response by finding new efficient and environmentally friendly antimicrobial drugs. Well-known drugs with environmentally friendly potential include metallic silver and silver-containing compounds, for example, in the form of silver salts, silver-ion-releasing molecules, and charged or neutral clusters of Ag nanoparticles. Their mechanical, physical, and chemical interactions with cells are so fast and intensive that pathogens hardly ever develop resistance. Not surprisingly, various silver species have found applications in washing mixtures, cosmetics, nutrient supplements, surgical blades, socks, shoes, food containers, teeth prostheses, mobile phones, catheters, wound dressings, color coatings, water purification systems, and more [1,2,3]. Silver itself and silver salts, including nanoparticles and other sources of ionic silver species (e.g., surfaces of oxides, zeolites, and other minerals) have been used since ancient times. They have been applied to various medical conditions—curing wounds, diarrhea, and other illnesses. The Food and Drug Agency (FDA) of the United States approved silver for medical applications in the 1920s [2,3].

The silver concentration in foods, on the other hand, is regulated in many countries. The European Commission determined the concentration of silver ions of 50 ppb as being safe in foods, but the FDA limited the levels of zeolite silver to 5% in food packages. The dissolution of silver and its quantity in the environment depends on the ambient conditions and vehiculum. One possible natural carrier of different silver forms are microporous aluminosilicate materials, namely zeolites.

Zeolites are crystalline alumino-silicate frameworks of interconnected TO_4_ (T = Si, Al) tetrahedra with a composition that can be given as Si_1−n_Al_n_O_2_xH_2_O. The addition of Al^3+^ to the structure results in a negatively charged skeleton. Positively charged, extra-framework ions, usually 1+ or 2+, are consequently included, to restore the neutrality of the structure. Such charge-compensating species could simply be protons, i.e., H^+^, NH_4_^+^, alkali, alkaline earth or transition metal ions, heavier metal ions, or charged clusters of atoms. The Si/Al ratio of the framework can vary from 1, as in zeolite A, also known as Linde Type A (LTA), up to infinitely high numbers, as in pure siliceous zeolites like Silicalite-1 from the mobile five (MFI) type. As the Si/Al ratio increases, there are fewer charge-compensating sites available in the framework, and consequently, there is a lower loading capacity for extra framework ions. The most up-to-date atlas of zeolite skeleton types lists 133 structures, all with different topologies [4].

Some zeolites occur in nature, and 40 different structures of those are known. One of the most studied and widespread naturally occurring zeolites, for instance, is clinoptilolite, which has found various key environmental applications as a drug carrier, a heavy metals removal agent in aqueous solutions, and as a low-cost green composite magnetic dye adsorption and removal reagent [5,6,7]. On the other hand, many new zeolitic structures have only been synthesized in laboratory conditions, usually through hydrothermal processes, with or without the assistance of organic and/or inorganic templates [8]. In all of the microporous aluminosilicate zeolite structures, monovalent ions, e.g., Ag^+^, are readily exchanged and the corresponding Ag forms are produced. Furthermore, different silver species, including charged clusters [9] and neutral nanoparticles [10], can be formed inside the carrier material’s pores and surfaces under controlled conditions. Thus, Ag-exchanged zeolites, including some post-treatments, represent a convenient system to study the antimicrobial activity of silver as the active species in different forms and concentrations. Presumably, this activity must depend on the readiness of the material to release the active species in solutions where it interacts with the target. The ion release characteristics are known to vary with the host scaffold topology and the types of ions in the surrounding environment, e.g., in K^+^-rich broth, Ag^+^ has been found to escape more readily from clinoptilolite than from zeolite A or zeolite X/Y [11]. Silver species are polarized by the strong electric fields inside the framework, and this leads to a strong attraction between Ag^+^ and the zeolite structure. In general, all zeolites show a high selectivity for Ag^+^, and as the Si/Al ratio for a particular framework increases, the selectivity for Ag^+^ tends to be higher [12].

In recent years, after a period of faded interest due to the wide use of organic medicines such as microbially produced antibiotics, the interest in silver-containing substances has attracted a constantly increasing amount of interest. Some excellent reviews and research works conducted in different labs are available, e.g., the one by Dutta and Wang (2019) [10]. When summarizing these studies in a few hundred works, consistent conclusions appeared to be difficult to draw. It seems there is an expected trend in the antimicrobial activity, increasing with the increase in the silver concentration, but exceptions also exist. Similarly, in various materials, either silver ions or silver nanoparticles have shown higher activities, while metallic silver particles are most often regarded as less active [10]. It should be mentioned that in many of the research works covered, a detailed and sound characterization of the state of silver was hard to achieve; moreover, it is well known that the relatively large size of monovalent silver ions, comparable to the size of the zeolite silicate rings, makes them quite mobile, thus experiencing a rather smooth potential surface inside the zeolite channels. This leads to a facile reduction and formation of either charged clusters or nanoparticles, under broad near-ambient conditions, during controlled treatments, e.g., vacuum and slightly elevated temperatures, reducing solutions. Accidental uncontrolled local overheating, however, may also occur during sample characterization procedures and/or storage, by irradiation with light, UV, higher-energy electromagnetic radiation e.g., X-rays, electron beams. For instance, Krishnani et al. (2012) [13] investigated the antimicrobial activity of Ag^+^-exchanged zeolite A (39.4 wt% Ag, assuming the pure ionic form of the silver species) against *E. coli*, *Vibrio harveyi*, *V. cholerae*, and *V. parahaemolyticus*. The minimum inhibitory concentration (MIC) for *E. coli* and *V. harveyi* was 40 µg/mL, while for *V. cholerae* and *V. parahaemolyticus*, the MIC was higher, at 50 and 60 µg/mL, respectively (after 48 h contact, 10^9^ CFU/mL). *V. cholerae* and *V*. *parahaemolyticus* have thicker cell walls and therefore require higher concentrations of Ag^+^–zeolite. The presence of ammonia was found to increase the antimicrobial activity of the Ag^+^–zeolite and was attributed to the toxicity of NH_3_.

Kaali et al. (2013) studied the ion exchange isotherms of single, binary, and ternary mixtures of Ag^+^, Cu^2+^, and Zn^2+^ with zeolite A (Zeomic). Ag^+^ shows a theoretical exchange of almost 100% [14]. This observation confirms the easy incorporation of silver into the zeolite material.

A lower Si-to-Al ratio often favors the auto reduction of silver and the formation of silver particles, which has been pointed out as the main reason for decreased antimicrobial activity as in the comparative study on Ag-X (Si/Al about 1.3) and Ag-Y (the same zeolitic topology but Si/Al about 2.5) [15]. This study found that Ag^+^-ion-exchanged zeolite X (9.8 wt% Ag) and Ag^+^-ion-exchanged zeolite Y (9.7 wt% Ag), both with micron-sized zeolite particles, show the following MIC values for *E. coli* and *Bacillus subtilis*: 300 µg/mL for AgX and 200 µg/mL for AgY (24 h exposure). For the yeasts *Saccharomyces cerevisiae* and *Candida albicans*, the MICs were 1000 µg/mL for both Ag-loaded zeolite X and Y (42 h exposure) [15]. The lower MIC value for bacteria treated with AgY, compared to AgX, was explained as arising from the metallic Ag (detected by spectroscopy) in AgX and the thinner cell wall. The thicker yeast cell envelope determines the higher concentrations of Ag^+^ zeolite needed for growth inhibition [15]. Similar results were obtained by Kędziora et al. (2018) [16], which appear to support the view that metallic silver particles manifest their antimicrobial activity by releasing ionic silver species, and the minimal inhibition concentration of Ag^+^ for Gram-negative bacteria is lower than that for Gram-positive bacteria because of their thicker cell walls.

In other scenarios, the cooperative effect of co-present counter ions like Zn^2+^ was studied by Ferreira et al. (2016) [17]. Their studies on bimetallic ZnAgY, tested for its activity against *E. coli* (CECT423), *B. subtilis* (4886), *C. albicans* (JGC 3456T), and *S. cerevisiae* (BY 4741), employed samples with different Ag/Zn loadings. ZnAgY with 3.03 wt% Zn and 6.04 wt% Ag were found to be most efficient, showing MICs of 100, 100, 300, and 300 µg/mL against *E. coli*, *B. subtilis*, *C. albicans*, and *S. cerevisiae*, respectively. For AgY with 9.70 wt% Ag loading, the comparable MIC values were higher, at 200 µg/mL for the bacteria and 1000 µg/mL for the yeast species, indicating enhanced activity with both ions present. The distribution of Zn^2+^ and Ag^+^ in the zeolite was found to be uneven, suggesting that Ag^+^ in particular is preferably found in the supercage sites, being large enough. A synergistic effect between Ag^+^ and Zn^2+^, however, was evident. Notably, and of great practical importance, the results of antimicrobial assays have been found to be reproducible even after two years of sample storage, which proves the stability of the zeolite [17].

Furthermore, the antimicrobial activity could be greatly influenced by the rate of the release of the silver species from the zeolite carrier. Parameters that could directly influence this property include the particle/crystallite size in which silver is accommodated, as well as possible surface morphology and/or the presence of particle structural hierarchy. The MIC and minimum bactericidal concentration (MBC) for Ag^+^-exchanged zeolite X, with different morphologies, have been investigated by Chen et al. (2017) [18]. Submicron aggregates of 100–700 nm, containing ~24 nm primary particles, were compared to ~2 µm particles. The nanostructured zeolite had a hierarchical structure with both micro- and mesopores. The amount of Ag^+^ loaded in both morphologies was similar, around 20–22 wt%. Not surprisingly, the Ag^+^ release characteristics in a flow cell (with Na^+^ eluent) showed a faster and larger amount of Ag^+^ release from the nanozeolite. However, in a 24 h test, micron-sized and nanozeolites showed MIC and MBC values against *S. aureus* (*MRSA*) of 16 and 32 µg/mL, respectively. In short-term experiments, e.g., 10 min, a faster Ag^+^ release from the nanozeolite was evident, with 400 µg/mL of the zeolite killing *MRSA* (10^8^ CFU/mL) in 3 min versus the 7 min needed for comparable concentrations of the micron-sized zeolite. Ag^+^ nanozeolite was ineffective in the inhibition of the *MRSA* biofilm; it appeared to aid in the film’s formation.

Cytotoxicity against human skin epithelial cells (WM-115) requires >128 µg/mL Ag^+^-hierarchical zeolite, while human skin fibroblasts (Detroit 551) and monocytes (U-937) require concentrations of 64 µg/mL to significantly reduce the viability. These cytotoxic concentrations have been found to be significantly higher (2–4 times) than the MIC/MBC concentrations of *S. aureus* (*MRSA*) [19]. However, all other authors present a better sensitivity of the eukaryotes and a better cytotoxicity of the nanocrystalline silver forms.

Contrary to the above findings from Chen et al. (2017) [18], Youssef et al. (2017) [20], who compared the zeolites analcime, faujasite, and zeolite A at both the micron- and nano-scales (~200 nm), found that during Ag^+^ exchange, the nanocrystalline zeolites degraded, but micron-sized zeolites were stable. Some very high levels of Ag^+^ were found in faujasite (48 wt%) and analcime (50 wt%) from the micron-sized zeolites and zeolite A containing 24.6 wt%. In those studies, the agar plate diffusion method showed antimicrobial activity in the following order, analcime > faujasite > zeolite A, against *S. aureus*, *P. aeruginosa*, *C. albicans*, and *A. niger*, but no difference in the antimicrobial activity was observed between micron-sized and nano-sized zeolites [20].

Yet again, to compare the activities of silver ionic species vs. nanoparticles, Lv et al. (2009) [21] investigated the bactericidal activity of two types of titano-silicate: one loaded with silver ions and another with silver nanoparticles, with approximately equal weight percentages for the silver species in both materials. The average size of the silver nanoparticles was found to be below 5 nm. It turned out that the highly porous titano-silicate, loaded with silver ions released silver ions faster and in higher concentrations, but they were less active towards the bacteria than the nanoparticle-loaded titano-silicate. Consequently, they concluded that silver nanoparticles show a stronger bactericidal effect than ionic species [21].

The modification of zeolite A (NaA) by impregnation with different concentrations of silver ions (Ag^+^) or silver nanoparticles (AgNPs) for antibacterial applications was also investigated by Jiraroj et al. (2014) [22]. The Ag^+^-NaA composites were prepared by ion exchange with different concentrations of silver nitrate solution (25–200 mg/L), while AgNPs were subsequently prepared by reducing the impregnated Ag^+^ with sodium borohydride. The NaA structure was not significantly affected by the addition of Ag^+^ or AgNPs. The distinct nature of the silver species in the materials under study was manifested in the measured UV spectra as a broad absorption peak at 394 nm, characteristic of the AgNPs in the AgNP-NaA composites that were missing in the Ag^+^-NaA material. The Ag^+^-NaA and AgNPs-NaA composites both showed dose- and time-dependent antimicrobial activity against the Gram-positive *S. aureus* and Gram-negative *E. coli* bacteria. In these studies, the ionic silver species, i.e., Ag^+^, showed a substantially higher antibacterial efficiency against *E. coli* than the AgNPs-NaA composites. Interestingly, for the same NaA zeolite materials with the highest silver loadings and at 3 h tests, the AgNPs-NaA composites were more effective than the Ag^+^-exchanged NaA against *S. aureus* [22].

The antimicrobial activities of low-silver-content samples, presumably in their ionic form in a zeolite ZSM-5 carrier, which is a low-Al-content-type zeolite (i.e., high silica), were studied by Lalueza et al. (2010) [23]. The low Al content of the zeolite naturally resulted in low Ag^+^ loadings, e.g., 0.2 wt%. Those Ag^+^ -ZSM-5 samples showed lg4 suppression within 24 h for *S. aureus 9213* (10^9^ CFU/mL); 25,000 ppm Ag^+^ was released in a 300 g/L^−1^ zeolite culture medium with 0.23 wt% Ag within the first 4–6 h of exposure. During longer timeframes (24 h), although more Ag^+^ was released, the biofilm formed (observed by SEM) around the zeolite hindered the migration of Ag^+^, which reduced the antimicrobial activity. These studies indicate that not only is the amount of silver is important, but so is the active species release kinetics as well as the susceptibility towards biofilm formation. Biofilm formation has also been investigated in other studies, addressing the applicability of silver-loaded materials for marine applications, namely as antifouling agents. For instance, Yee et al. (2015) [24] reduced Ag^+^-ZSM-5 (1–5 µm zeolite with Ag content 0.8–10 wt%) with citrate to obtain AgNPs (~1.48 nm). The adherent bacterial biomass of *H. pacifica*, a common marine-fouling organism, decreased by 81% in the presence of 10 wt% Ag^+^. They observed that the formation of biofilm was greatly inhibited at higher Ag loadings of the zeolite, up to the highest achievable loadings of 10 wt%. Ag zeolite also inhibited the growth of other marine microalgae [24].

Singh et al. (2015) [25] noted that dispersed Ag metal on zeolite crystals can react with H_2_O_2_, producing O_2_, which causes these crystals to move in a liquid medium. These Ag metal zeolites can be ion-exchanged with Ag^+^ and have been found to exhibit antibacterial properties against *E. coli*. This self-induced movement resulting in increased contact with the bacteria is of interest in this study and could be used to understand the antimicrobial activity of the material, as the microorganisms form H_2_O_2_ during their metabolic activity [25].

As seen from the above retrospective review of previous works on the bactericidal activity of silver species, there is not yet agreement on the answers to the primary questions regarding which is the more active type of silver species, nor on the dependency of this activity on the silver concentration. No preferable type of active species carrier has been put forward either. Hence, clear recipes for preparing an efficient and reproducible antibacterial material based on silver or other metal ions or other metal species remain hard to devise.

The generation of reactive oxygen species (ROS) and prooxidant activity is an important biomarker of antibacterial activity and, on the other hand, is also demonstrative of the antioxidant effects of synthesized materials. It is very important to measure the kinetics of free radical generation in order to estimate the pro- and antioxidant properties of a material [26,27], keeping in mind that most materials change drastically their properties at the nanoscale or depending on the pH level. This information is very important to elucidate the mechanisms of action of new materials on micro- and macro-organisms.

The chemiluminescent assay is a fast and extremely sensitive method for use in such studies. It is applied to monitor the dynamics of free radical generation and to determine the prooxidant/antioxidant activity of various substances. According to this method, probes are applied that enhance the luminescent emission of these substances in order to achieve comparable signals. These reactions emit light within the range of 480–580 nm and can be harnessed to assess the quantum yield of the excited state products generated [27,28].

In the present work, we studied the bactericidal activity of the silver species originating from synthesized materials in their two main primary forms: silver nanoparticles with crystallite sizes in the range of 5–10 nm and monovalent silver ions, both contained in various microporous zeolite materials. The redox activity of these materials was tested in free radical oxidation reactions at pH 7.4 (physiological) and pH 8.5 (optimal) in chemiluminescent model systems. Finally, the environmental safety of all studied materials was tested via an acute toxicity test against young *Daphnia magna*.

## 2. Results and Discussion

**Figure 1:**
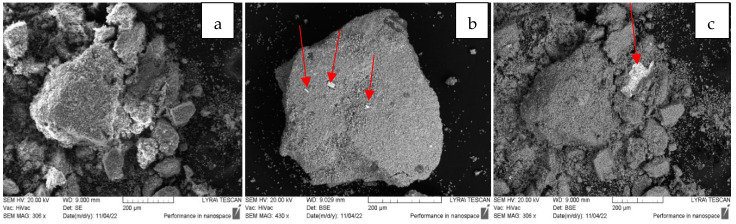
AgX and Ag_84_Na_2_[(AlO_2_)_86_(SiO_2_)_106_] × H_2_O, with a nominal 35%wt silver content, which was determined to be about 36% by EDX, and was up to 68% within the area of the large silver particle seen in the micrograph: (**a**) the as-received AgX material, sample AgXas; (**b**) the vacuum/heat reduced AgXcl; (**c**) the regenerated AgXrc material.

The main physicochemical properties of the studied materials are summarized in Table 1. The silver chemical content was evaluated by the EDX analyses. The silicon-to-aluminum ratio is given as provided by the zeolite suppliers. The MFI and FAU topologies were verified by the XRPD analyses. The corresponding powder diffraction patterns are shown in Figures S1–S5, confirming that the main zeolitic structures remain intact after all treatments. However, a metallic silver FCC-phase was detected due to the presence of the corresponding (111) and (200) reflections in the PXRD patterns of both the reduced and reactivated silver-exchanged zeolite X materials, whereas the silver-exchanged ZSM-5 zeolites showed a pure MFI phase. Further inspection of the materials by SEM/EDX confirmed that some large metallic silver particles are present in the AgX materials (Figure 1).

**Figure 2:**
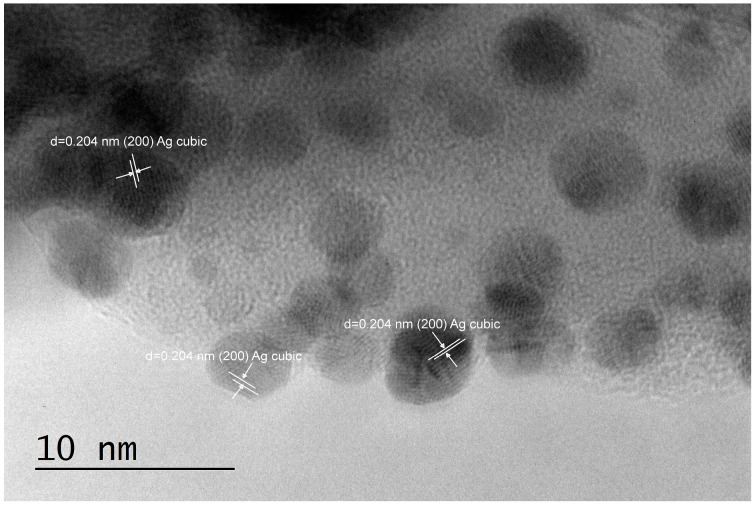

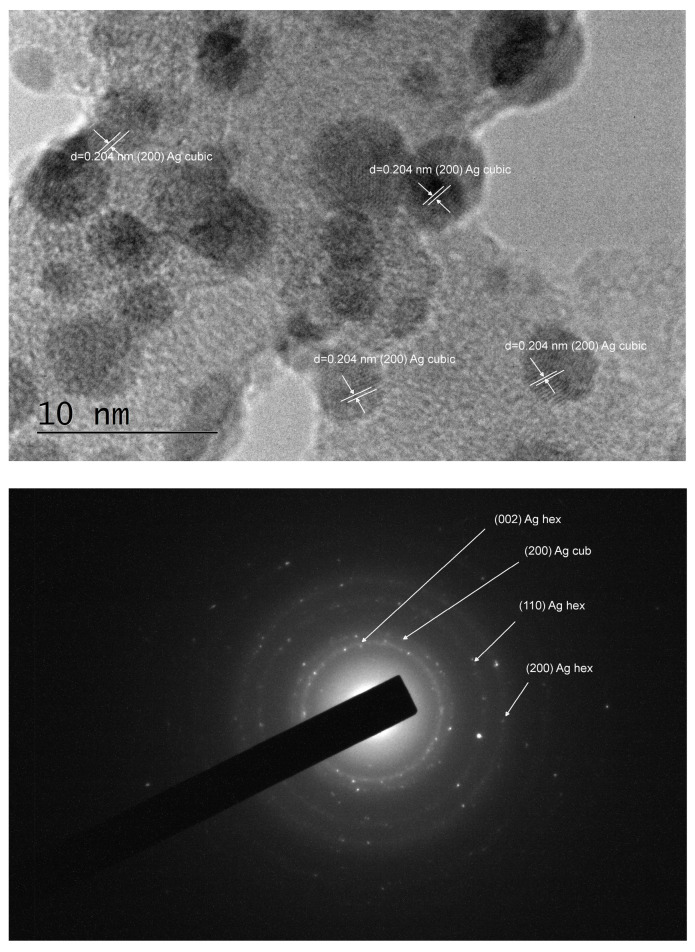
TEM images and analyses of the reduced Ag-exchanged zeolite X, sample AgXcl.

**Figure 3:**
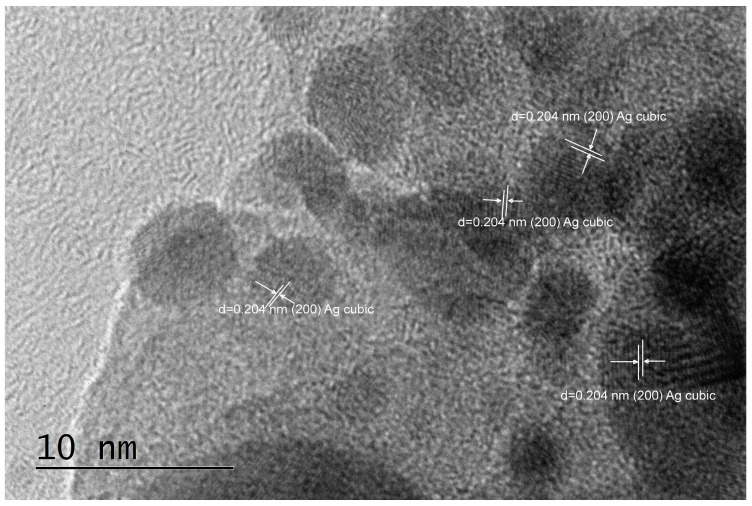

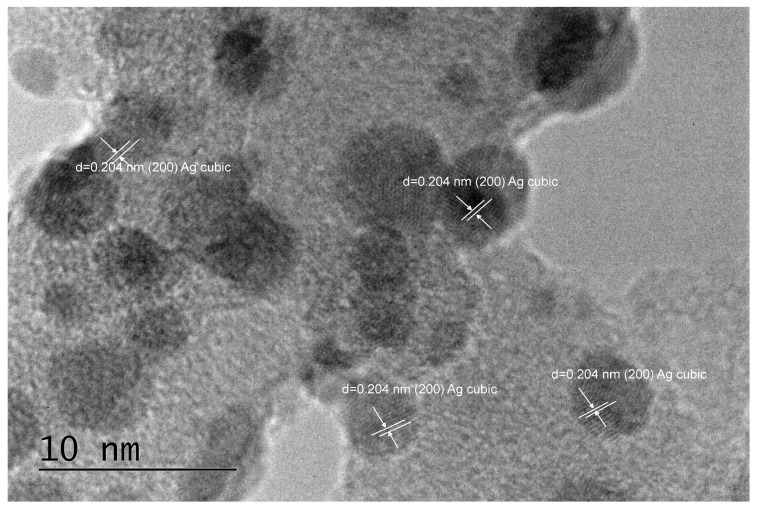
TEM images and analyses of the regenerated Ag-exchanged zeolite X, sample AgXrc.

**Figure 4:**
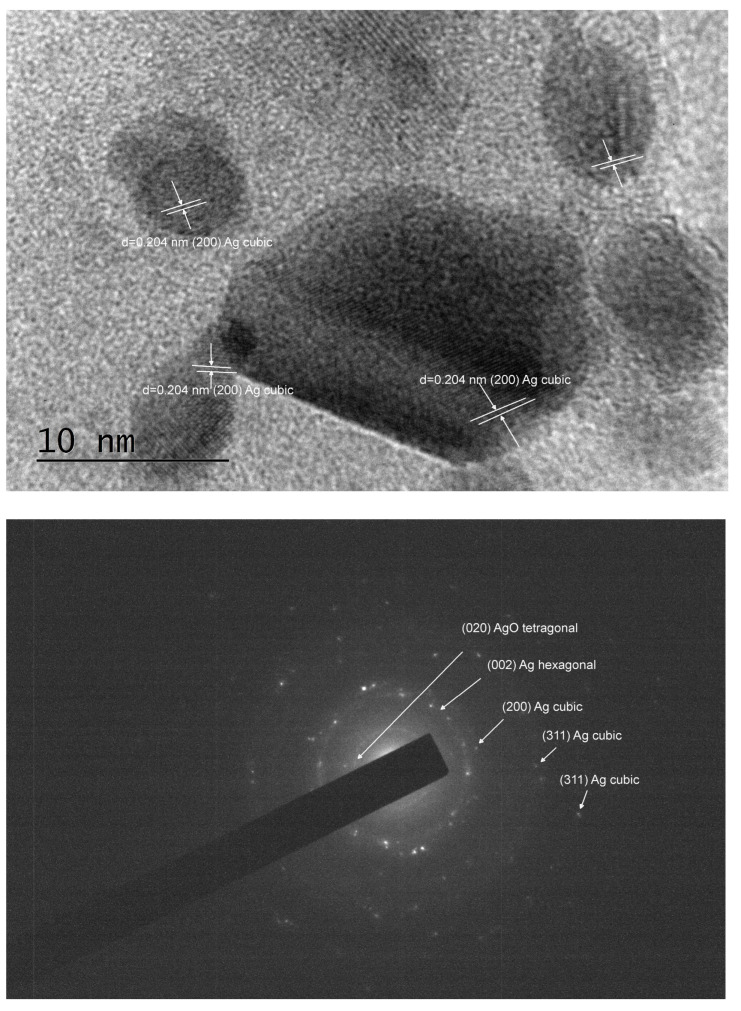
TEM images and analyses of the as-received Ag-exchanged zeolite X, sample AgXas, where some nanocrystalline silver particles could also be observed.

A deeper insight from TEM imaging (Figure 2, Figure 3 and Figure 4) revealed that silver nanoparticles, 5–10 nm in diameter, are abundant in the reduced AgX material; some were still found in the regenerated material, but nanocrystalline particles of similar sizes were also found in the as-received AgX zeolite. A minor amount of hexagonal-phase silver could only be detected in some of the TEM images and only in the AgX zeolite sample (Figure 2 and Figure 4), but not in the sample of the same zeolite material regenerated in air. We must also point out that the electron beam in microscopy experiments, under vacuum, locally heats up the samples under investigation, and this also might be sufficient to produce some reduced silver species, e.g., charged clusters and/or metallic nanoparticles.

**Figure 5:**
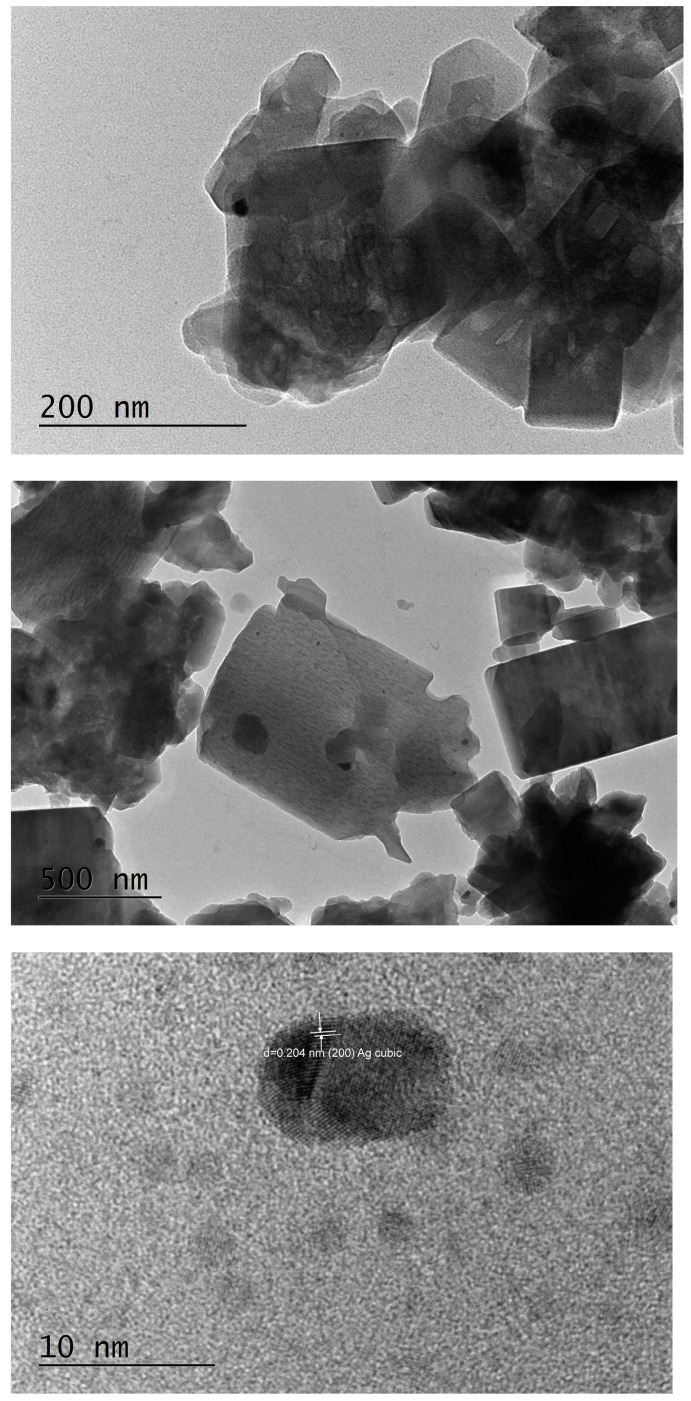
TEM images of SA12-Ag, 3.5%wt silver loading material, in which some scarce nanocrystalline silver was found too.

Notably, during the PXRD experiments with the as-received AgX material, we observed a dark stripe on the sample after the measurements, exactly on the irradiated sample area, which is evidence that the intense X-ray beam causes silver reduction under ambient conditions. This should not be surprising, considering that silver exchange procedures are usually performed in the dark and samples, when stored for prolonged periods, are also kept in the dark. To avoid this effect, at least upon visual inspections, the X-ray tube’s current was reduced down to 20 mA, and the reported diffraction patterns were measured for a total exposure time of about 1 h. However, one must generally bear in mind that, in zeolitic materials, isolated silver ionic species are relatively easily reduced to larger size species, such as metallic nanoparticles. Hence, experimental methods that involve the use of intense light beams directed to the sample and techniques involving uncontrolled local overheating of probed sample regions may not be considered truly nondestructive. These effects were not as pronounced for low silver contents, e.g., in high-silica zeolites like the ZSM-5 zeolites, in which we did not detect silver nanoparticles under equivalent characterization conditions. The corresponding PXRD patterns are shown in Figures S1–S5. However, some scarce silver nanoparticles were observed in the highest silver concentration, higher Al-content ZSM-5 zeolite sample SA12-Ag (Figure 5). The cause for the appearance of these metallic silver nanoparticles is unclear at present. It may be due to the electron beam in the TEM microscope, but may have also formed during the exchange procedure in the Ag-solution.

**Figure 6:**
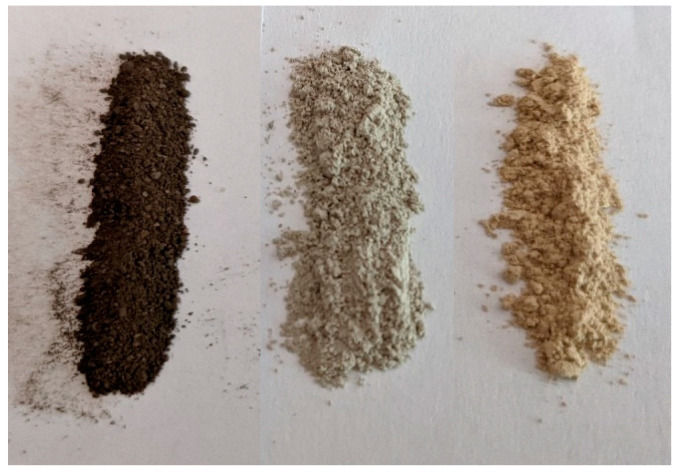
Visual appearance of the three AgX powder materials: from the left to the right AgXcl, AgXas, and AgXrc.

As seen in the TEM images above (Figure 2, Figure 3 and Figure 4), all of the AgX samples contained metallic nanoparticles, which were quite abundant in the black AgXcl reduced material, with a particle size predominantly under 5 nm. As clearly indicated by the visually observed corresponding powders’ colors (Figure 6), the as-received material must contain very few such metallic particles in contrast to the black AgXcl material (Figure 6). We spotted some in the synthesized material (Figure 4), of even larger than 5 nm size, e.g., about 10 nm. Although not abundant, such silver nanoparticles could even be found in the ZSM-5 material, with 3.5%wt silver-exchanged material. Evidently, the presence of silver nanoparticles in most silver exchanged materials is confirmed.

In our case, apart from the different zeolitic carrier material types, the investigated materials can also be distinguished by their silver content and form, as described in the third column of Table 1. Thus, we distinguish our materials mainly by the total silver content, once it had been controlled by the different Si-to-Al ratios of the X and MFI zeolite topologies, then by the different exchange protocols of the ZSM-5(MFI) materials, and third by the dominant presence of silver species: (i) mainly silver nanoparticles in the AgXcl material; (ii) mainly Ag^+^ in the as received AgX material (AgXas) with a minority of silver nanoparticles; (iii) Ag^+^ in the AgXr material, with abundant silver nanoparticles, including all three types with an equal total silver content; and (iv) Ag-exchanged ZSM-5 zeolites with different Ag contents, between 1 and 3.5%wt, that were an order of magnitude lower than that in the X-type zeolite samples, which led to a much larger separation of Ag ions within the framework and hence a much lower chance for silver reduction and nanoparticle formation. The corresponding PXRD patterns in Figures S3–S5 show only the pure MFI phases for all of the ZSM-5 materials, with no sign of metallic silver, in contrast to those for the AgX type of materials (Figures S1 and S2).

**Figure 7:**
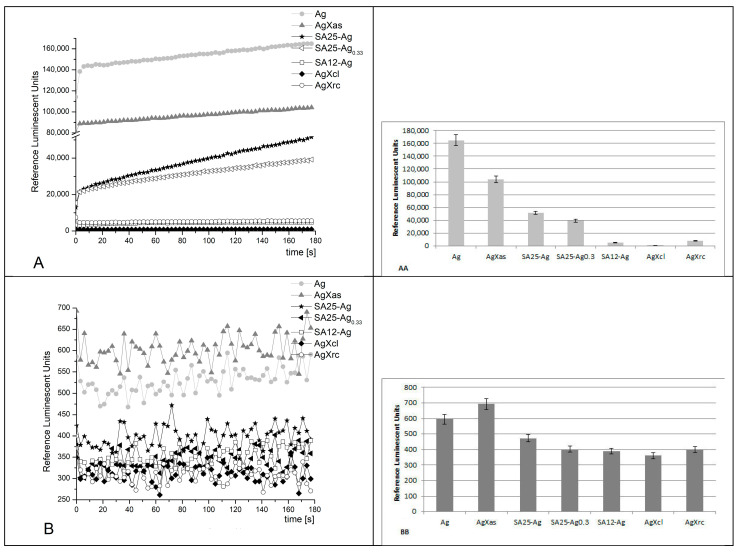
Fenton’s chemiluminescence induced by ·OH and ·OOH radicals at pH 8.5 (**A**,**AA**) and pH 7.4 (**B**,**BB**), and the effect of the synthesized materials (*p* ≤ 0.05); reagents: FeSO_4_ (5 × 10^−4^ M) + H_2_O_2_ (1.5%) + lucigenin (10^−4^ M) at 37 °C.

The chemiluminescent assay produced the following results:

- System I (Figure 7)

The Fenton’s reaction between Fe^2+^ ions and H_2_O_2_ produces highly reactive, short-living ^·^OH and ^·^OOH radicals. Usually, the achieved chemiluminescent emission is much higher than the one from other mixtures:

(1) Fe^2+^ + H_2_O_2_ → Fe^3+^ + ^·^OH + ^−^OH
(2) Fe^3+^ + H_2_O_2_ → Fe^2+^ + ^·^OOH + H^+^

The registered signals from this reaction, with the application of various synthesized materials, showed that, at pH 8.5 all reactions possess similar kinetics showing a plateau, which slightly increases with time. This is indicative of the silver species released in the solution with time. The sample containing control Ag nanoparticles (30–50 nm) emitted the highest signal (100%) as expected, with a maximum RLU of ~165,000. The AgXas sample showed about a 40% decrease in emission, followed by SA25-Ag at 70%, SA25-Ag_0.33_ at 77%, SA12-Ag at almost 98%, and AgXcl at 99%. The AgXrc sample exhibited a 95% decreased emission in comparison to the control Ag nanoparticles.

At pH 7.4 (physiological), the kinetic curves were not smooth and had various fluctuations. AgXas demonstrated a prooxidant activity, that was 16% higher than the control Ag nanoparticles. All the other tested materials exhibited a suppressed emission in comparison to the control activity: SA25-Ag—20%; SA25-Ag_0.33_—33%; SA12-Ag—35%; AgXcl—40%; AgXrc—33%.

The reactions are indicative of and comparative with the level of silver ions or nanoparticles released by the various samples and their effect on the free radical generation in comparison to the control with the pure Ag nanoparticles.

**Figure 8:**
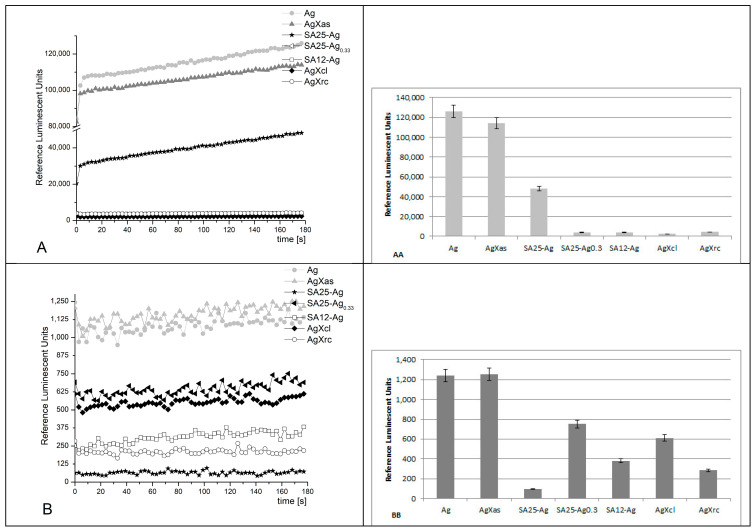
Chemiluminescence induced by H_2_O_2_ at pH 8.5 (**A**,**AA**) and pH 7.4 (**B**,**BB**) and the effect of the synthesized materials (*p* ≤ 0.05); reagents: H_2_O_2_ (1.5%) + lucigenin (10^−4^ M) at 37 °C.

- System II (Figure 8)

In this system, a strong oxidant, hydrogen peroxide, serves both as an oxidizing agent and an ROS. At alkaline pH, the kinetic curves were smooth, and the signal in all the reactions was increasing in time. The mixture, containing pure silver nanoparticles (control), emitted most light, as expected. All of the other tested samples demonstrated lower signals as follows: AgXas—10%, SA25-Ag—more than 60%, SA25-Ag_0.33_—97%, SA12-Ag—97%, AgXcl—99%, and AgXrc—97%.

At pH 7.4, AgXas was as active as the control. All of the other tested samples presented a lower luminescent emission in comparison to the control with pure Ag nanoparticles, as follows: SA25-Ag—more than 93%; SA25-Ag_0.33_—40%; SA12-Ag—70%; AgXcl—50%; AgXrc—78%. In general, the registered emissions very slightly increased with time, showing the release of reactive Ag species.

**Figure 9:**
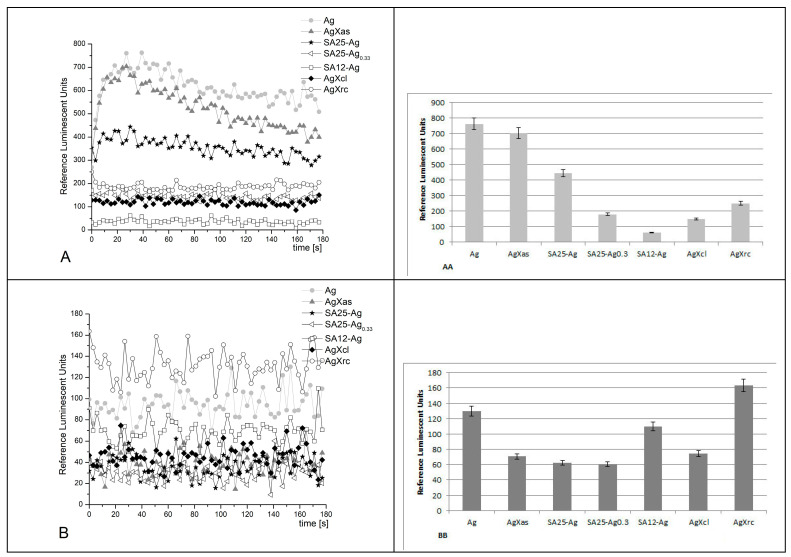
Chemiluminescence induced by O_2_·^−^ radicals at pH 8.5 (**A**,**AA**) and pH 7.4 (**B**,**BB**) and the effect of the synthesized materials (*p* ≤ 0.05); reagents: NADH (10^−4^ M) + PhMS (10^−6^ M) + lucigenin (10^−4^ M) at 37 °C.

- System III (Figure 9)

The O_2_^−^ generation in this system occurred according to the following chemical scheme [29,30]:

(1) PhMS + NAD.H + H^+^ → PhMS.H_2_ + NAD^+^
(2) PhMS.H_2_ + PhMS → 2PhMS.H^.^
(3) PhMS.H^.^ + O_2_ → PhMS + O_2_^−^· + H^+^

The superoxide radical produced is very short living and aggressive.

At pH 8.5, all the tested materials demonstrated an activity against that ROS lower than the control, as follows: AgXas—an 8% reduction; SA25-Ag—more than 40%; SA25-Ag_0.33_—77%; SA12-Ag—more than 90%; AgXcl—80%; AgXrc—almost 70%. A higher level of oxidation was observed at the beginning of the reactions, and the emitted light decreased with time. The neutralization of that ROS was very effective for all the tested materials.

At pH 7.4, the observed properties were maintained for all materials except AgXrc. It was oxidized 25% more than the control consisting of pure Ag nanoparticles, indicating that it was more efficient in the release of silver species. All the other tested materials suppressed the generation of O_2_^.–^ radicals: AgXas—46%; SA25-Ag—53%; SA25-Ag_0.33_—54%; SA12-Ag—16%; AgXcl—more than 40%. Despite the significant fluctuations observed, the registered effects were maintained with time.

In conclusion, AgXas and AgXrc appear to be the best candidates and rivals of the pure Ag nanoparticles in such ROS-generation reactions, especially within physiological conditions (pH 7.4/37 °C), showing a prooxidant activity. That result can explain their strong bactericidal effects.

**Figure 10:**
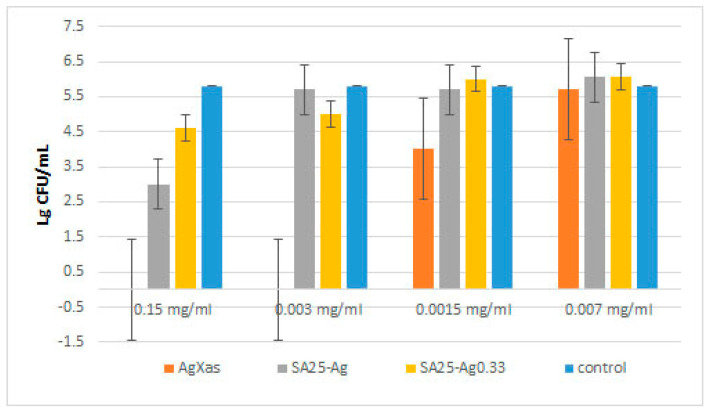
Results for *E. coli* *ATCC 25922* at the 4th h of impact of zeolites AgXas, SA25-Ag, and SA Ag_0.33_.

The antibacterial function of the silver-doped materials was studied by monitoring the growth-inhibitory effect on *Staphylococcus aureus* *ATCC 25923* and *Escherichia coli* *ATCC 25922*. Figure 10 presents the results obtained from a screening using the most probable cell number method. It appeared that the well diffusion method was not suitable for studying zeolitic materials because agar hindered the diffusion of nanoparticles and the sterile zones around the wells were not clear. According to the well agar diffusion method, only the dispersion of AgXas diffused into the culture medium; around the 6 mm diameter well, a sterile zone with an average value of 12.5 mm was measured for *E. coli*. This indicated a bactericidal effect of the dispersion at a concentration of 5 g/L^−1^. At lower concentrations, sterile zones were not detected. According to the spot test, with the same bacteria, a strong bactericidal effect on *E. coli* was observed, from 5 g/L^−1^ to the lowest concentration tested, 0.15 g/L^−1^. The agar medium did not allow for the diffusion of zeolite dispersions. For that reason, the spot test (based on direct contact with bacterial cells) appeared to be more reliable for the demonstration of the bacterial toxicity (results are not presented).

The results obtained after counting the surviving cells on the inoculated plates after treatment with different types of Ag-loaded zeolites in liquid medium are presented in Figure 10 and Table 2.

As can be seen from Figure 10, AgXas exhibited the strongest effect on Gram-negative bacteria, as it killed all bacteria at concentrations of 0.003 g/L^−1^ and above. The MIC of AgXas against *E. coli* was 0.0015 g/L^−1^. The other zeolites had almost no effect at the same concentration. The other strong bactericidal effect was observed for SA25-Ag. Perhaps the silver ions were more efficient. On the other hand, a higher Si-to-Al ratio seemed to be more beneficial, due to a higher release rate and concentration of silver ions in the media.

The other silver-loaded zeolites, SA25-Ag, AgXcl, and AgXrc, both fresh and old dispersions, manifested a bactericidal effect at a concentration of 2.5 g/L^−1^. The zeolites with silver nanoparticles (those, originating from AgXcl or AgXrc, which also releases silver ions), possessed the strongest bactericidal effect in comparison to other zeolitic materials presented in Table 2. It demonstrated a 99% bactericidal effect on *E. coli* at a concentration of 0.25 g/L^−1^ too. This is more proof that the silver ions appear to be the most active silver species against bacteria.

As can be seen from Table 3, after 24 h the zeolite material AgXas manifested a 99.999% bactericidal effect on *E. coli* *ATCC 25922* at a concentration of 0.15 g/L^−1^. The SA25-Ag material demonstrated the same bactericidal effect while the same carrier zeolite material, loaded with fewer silver ions, i.e., 1.2%wt vs. 2.5%wt, as shown in Table 1, showed only an inhibitory effect on the tested Gram-negative bacteria.

As presented on Table 4, the freshly prepared dispersions of SA12-Ag, AgXcl as well as the old ones, that were stored in a refrigerator for 2 months, manifested a bactericidal effect at a concentration of 0.025 g/L^−1^. SA12-Ag exhibited only a 10% inhibitory effect against *E. coli* *ATCC 25922*, at the same concentration. The SA12-Ag material demonstrated a bactericidal effect on *E. coli* at a concentration of 2.5 g/L^−1^. The freshly prepared dispersion AgXrc produced a bactericidal effect at a concentration of 0.25 g/L^−1^. After storage in a refrigerator for 2 months, the same dispersion lost its activity, with a bactericidal effect of 99.99% at a concentration of 2.5 g/L^−1^. In addition to the dose/concentration-dependent effect, a time-dependent effect was also observed for samples SA12-Ag(I) fresh, AgXcl fresh, AgXcl old, and AgXrc fresh (when compared between the fourth and twenty-fourth hours on *E. coli* *ATCC 25922*).

Clearly, the zeolites released both silver nanoparticles and silver ions, which leached from them quickly; their effect was strong during the first 4 h after interaction with the bacterial suspension. After 24 h, the bacterial cells presumably released proteins and other cell substances during their lysis, which decreased the effect of leached silver species from the zeolite materials.

*Staphylococcus aureus* is a Gram-positive bacterium with almost 80 layers of peptide-glucan in its cell wall. The thickness of the cell wall determines the resistance of the bacteria to nanoparticles and other mechanical injuries. In our experiment, 0.15 g/L^−1^ of AgXas killed all living *S. aureus* cells, which was same as in the case with *E. coli* (Table 5). The results with other zeolitic materials loaded with silver ions were similar to those obtained with Gram-negative bacteria. The sample with less loaded silver, SA25-Ag_0.33_, showed only four times fewer living bacterial cells in comparison to the control at the 4th h of treatment (Table 5). This result proved the importance of the concentration of eluted silver ions for the antibacterial effect of the material.

The results shown in Table 6 demonstrate the strongest bactericidal effect of the silver nanoparticle-containing zeolite AgXcl, at a concentration of 0.25 g/L^−1^, on *S. aureus* *ATCC 25923* [31,32]. The most probable mechanism of action of the observed bactericidal effect is by the mechanical destruction of the cell wall of bacteria that are negatively charged by the leached nanoparticles; nanoparticles also interact with the water envelope around bacteria and the eluted silver ions that enter through the bacterial membrane in the cytoplasm are positive ions (I) [1,2,3]. The silver in these clusters may be the most concentrated material that releases more silver ions in comparison to other zeolite composites.

A time-dependent antibacterial effect was also seen when the old water dispersions of zeolite materials lost their effect after storage in a refrigerator, despite sonication immediately before the second test. The results could be important when these materials are produced in large quantities, and the expiration date should be considered.

**Figure 11:**
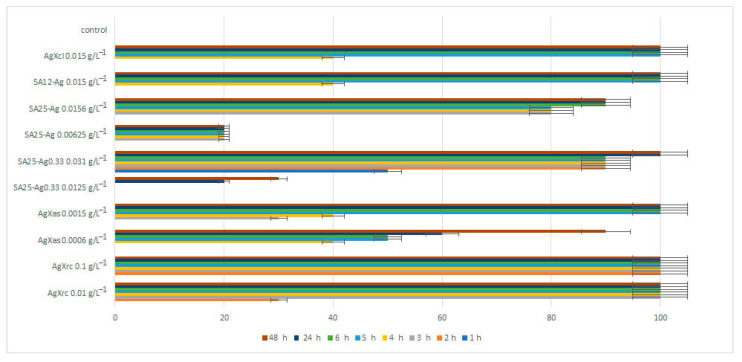
Interactions of Ag-loaded zeolites with *Daphnia magna*, given as lethality percentage.

The findings about the effect of the synthesized zeolite-based materials on the lethality of *Daphnia magna*, evaluating their ecotoxicity, are shown on Figure 11.

Water dispersions from silver-loaded zeolite materials were tested immediately after sonication on young *Daphnia magna* individuals at four different concentrations (not all presented in the figure below).

Figure 11 shows that the pure zeolite did not cause lethality against the young *Daphnia* according to the acute tests. AgXas and SA12-Ag showed a 90% lethality at the end of 48th h. SA25-Ag_0.33_, at a concentration of 0.0125 g/L^−1^, had a 30% lethality. SA25-Ag, at a concentration of 0.00625 g/L^−1^, had only a 20% lethality against *Daphnia magna* until the 48th h. AgXrc, AgXcl, and SA25-Ag were toxic and caused 100% lethality from the 2nd to the 4th h of the experiment. Generally, *Daphnia magna* were much more sensitive to the silver-loaded zeolite materials than the tested bacteria, presumably due to the slightly acidic pH of the water (6.5). These materials should not be released into the environment without control.

In general, silver nanoparticles exhibit strong antibacterial properties due to their small size. This small size increases the surface-to-volume ratio, enhancing the reactivity of the nanoparticles, which makes them effective against biofilm growth and microbial activity. They release silver ions over time, offering a sustained antimicrobial effect. However, the aggregation of nanoparticles can reduce their surface area, diminishing their reactivity and efficiency [16,33,34].

Silver ions (Ag^+^) are also known for their strong antimicrobial effects, but their interaction with microbes tends to be more straightforward. They bind to the cellular components of bacteria, disrupting essential functions and starting the generation of ROS. However, they may have a lower efficiency compared to the silver nanoparticles due to their smaller size and predisposition to form complexes with other substances in water, such as chloride ions, which can reduce their availability and antimicrobial action [1,2,3,16,25,33,34,35].

In summary, the silver nanoparticles provide a more sustained and potentially more effective antimicrobial action compared to silver ions, but they are resource-intensive and pose concerns regarding aggregation and environmental impact. Silver ions, while effective, are limited by their tendency to form inactive compounds and may require higher concentrations to have the same level of efficacy as silver nanoparticles [16,31].

The release of Ag species at bactericidal concentrations into the environment should be avoided to prevent irreversible changes in the food chain and ecosystems. The synthesized silver-loaded zeolite materials could have a wide range of potential applications due to their unique properties, including antimicrobial activity, Ag species exchange capacity, high surface area, and possible catalytic properties. These results support their applications in air and water filtering and other similar systems, coatings for medical devices, wound dressings, fabrics with antimicrobial properties, soil additives, food packaging and preservation, construction materials, etc.

## 3. Materials and Methods

### 3.1. Materials

Ag-exchanged zeolite X (AgX) was purchased from Sigma-Aldrich, Steinheim, Germany, with a nominal composition of Ag_84_Na_2_[(AlO_2_)_86_(SiO_2_)_106_] × H_2_O, and a granular form with 35%wt silver content. The samples for antimicrobial and all other tests were prepared by gently grinding the material down to powder form. The as obtained powder was split into three samples: (1) AgXas—powder form of the as received material. (2) The rest of the powder was subjected to vacuum heat treatment in a vacuum oven up to 200 °C for 4 h and pressure down to about 1 mbar, measured at the exhaust line of the oven. This led to a visible change in the powder’s appearance with a color change from ivory to black (sample AgXcl). (3) Part of this sample, AgXcl, was heated under a dry air flow in a furnace at 400 °C for 4 h, resulting in a color change back to ivory-reddish, designated as AgXrc. ZSM-5 materials, in their ammonium form, with silicon-to-aluminum ratios of 25 and about 12, were purchased from Zeolist International, Conshohocken, PA, USA (CBV2314G and CBV5524G). These were converted into their protonated forms by heating in air at 550 °C for about 2 h and labeled as SA12 (for Si-to-Al = 12) and SA25 (for Si-to-Al = 25) further in the text. The silver-exchanged samples were produced by wet exchange from 0.05 M silver nitrate water solutions at room temperature overnight in a dark room. The corresponding silver-exchanged powders were filtered out of the solutions, washed twice by stirring again in deionized water, and filtered. The resulting silver-exchanged materials were dried overnight in air at 80 °C. Ag-ZSM5 samples were white just like their original ammonia and H forms. Selected samples were characterized with SEM (LYRA I XMU, Tescan (Brno-Kohoutovice, Czech Republic)) and EDX (Quantax, Billerica, MA, USA) for agglomerate morphology and chemical composition. The zeolites and, eventually, the presence of silver metallic phases were checked by X-Ray Powder Diffraction (XRPD) using a URD-6 diffractometer, Freiberger Präzisionsmechanik GmbH, Rich. Seifert & Co. GmbH & Co. KG, Freiberg, Germany, using a Cu radiation source. Transmission Electron Microscopy (TEM) was performed with the materials and silver cluster/nanoparticle formation was anticipated to show the presence of metallic silver particles using a high-resolution, STEM JEOL JEM 2100 instrument with JEOL, Akishima, Japan. TEM images were recorded using a CCD camera (GATAN Inc., Pleasanton, CA, USA).

The following materials used for the chemiluminescent assay were purchased with a high purity. Those were iron sulfate (p. a.) (Merck, Darmstadt, Germany), hydrogen peroxide (30%) (Merck, Germany), phenazine methosulfate (PhMS) (N-methyldibenzopyrazine methyl sulfate salt) (p. a.) (Merck, Germany), lucigenin (bis-N-methylacridinium nitrate) (p. a.) (Sigma-Aldrich, St. Louis, MO, USA), β-nicotinamide adenine dinucleotide, reduced form (p. a.) (NAD.H, Boehringer, Ingelheim am Rhein, Germany), dimethyl sulfoxide (p. a.) (DMSO, Sigma-Aldrich), buffers at pH 8.5 and pH 7.4 (Sigma-Aldrich), and silver nanoparticles (99%, 30–50 nm) (Houston, TX, USA, US Research Nanomaterials Inc.). All chemicals were used as purchased.

### 3.2. Chemiluminescent Assay

We employed the luminescent method to study the effect of the synthesized silver materials on the kinetics of free radical oxidation reactions using activated chemiluminescence and a probe, lucigenin, in three model systems [35].

Usually, a medium with a higher alkalinity favors free radical generation and enables the achievement of reliable measurements and comparable differences. Two different media were tested: pH 7.4 and pH 8.5, physiological and alkaline, and optimal. Three ex vivo model systems were implemented [35].

The first model system is called Fenton’s. It generates ·OOH and ·OH radicals: it contained 0.2 mol of sodium hydrogen phosphate buffer, with the selected pH, Fenton’s reagent: FeSO_4_ (5.10^−4^ mol)—H_2_O_2_ (1.5%), lucigenin (10^−4^ mol), and 0.025 g/L^−1^ of the tested material.

The second model system contained the strong oxidant hydrogen peroxide (H_2_O_2_): 0.2 mol sodium hydrogen phosphate buffer, with the chosen pH, H_2_O_2_ (1.5%), the chemiluminescent probe, lucigenin (10^−4^ mol), and 0.025 g/L^−1^ of the tested material.

The third model system generated superoxide radicals (O_2_·^−^) in the reaction between NADH and PhMS [36]: it contained 0.2 mol of sodium hydrogen phosphate buffer with the chosen pH, NAD.H (10^−4^ mol), phenazine–metasulfate (10^−6^ mol), lucigenin (10^−4^ mol), and 0.025 g/L^−1^ of the tested material.

The control samples contained 30–50 nm silver nanoparticles (0.025 g/L^−1^). The reactions were monitored for 3 min, every 3 s, at 37 °C (physiological). All tested materials were sonicated for 60 min before application. All experiments were performed in triplicate.

### 3.3. Microorganisms

The antimicrobial effect was tested on Gram-positive and Gram-negative bacteria, *Escherichia coli* *ATCC 25922* and *Staphylococcus aureus* *ATCC 25923*, which were both supplied by the National Bank of Industrial Microorganisms and Cell Cultures (NBIMCC), Sofia, Bulgaria. The test microorganisms were grown in nutrient broth (NB Conda, Madrid, Spain) at 37 °C and 180 rpm for 24 h with 2 sub-cultivations to reach the exponential growth phase. The microbial density of the cultures was adjusted to 0.5 according to the McFarland standard and used for the antimicrobial tests.

### 3.4. Antimicrobial Activity

Two classical methods were used to study the antibacterial effect of the synthesized nanoparticles: spot and well diffusion tests in agar medium. Aliquots of 100 µL microbial suspension in exponential phase with optical density 0.5 MacFarland were randomly spread on solid medium (Nutrient agar, Conda, Spain) for both tests.

Spot test: 10 µL drops of the tested nanomaterial were put on inoculated agar. The used concentrations for spots were 3.0 g/L^−1^, 1.5 g/L^−1^, 1.0 g/L^−1^, and 0.75 g/L^−1^.

Well diffusion test: Wells with a diameter of 6 mm were cut using a stopper borer and filled with 50 µL solutions of nanozeolites in concentrations of 1.0 g/L^−1^, 0.5 g/L^−1^, 0.3 g/L^−1^, and 0.1 g/L^−1^. For both methods, the plates were left for 2 h at 5 ± 1 °C to afford the diffusion of the dispersions, and after that cultivated for 24 h at 37 °C. The obtained sterile zones were measured with 0.5 mm accuracy. All experiments were performed with two replicates, and the results were presented as the mean value of two experiments.

The determination of minimum inhibitory (MIC) and minimum bactericidal (MBC) concentrations of the nanomaterials was defined after preparing consecutive two-fold dilutions in liquid medium, and after 24 h and 48 h of cultivation at 37 °C, aliquots of bacterial suspension were inoculated in agar plates to determine the amount of bacteria that survived. The colony-forming bacteria is calculated using the formula:

N = A × 10^n^/0.1

where A is the mean number of colonies from the two replicates counted on solid medium for a given dilution, 10^n^ is the corresponding dilution from which the inoculum was taken, and 0.1 is the amount of inoculum on one Petri dish.

In order to determine the stability and effectiveness of the zeolite dispersions in water, time-dependent experiments were performed. The dry materials were stored at room temperature in the dark, and water dispersions were stored in a refrigerator at 4–6 °C for 1–2 months and tested again. They were, respectively, labeled as “fresh“ and “old” in the tables with results.

### 3.5. Environmental Toxicity by Testing the Lethality of Daphnia magna

Acute ecotoxicity tests were performed by using *Daphnia magna* for all types of materials that were applied in several concentrations, with three replicas of 10 young individuals for each substance and concentration, with the appropriate control. The concentrations varied from 0.0001 g/L^−1^ to 0.1 g/L^−1^. The experiments were conducted at ambient temperature 22 ± 1 °C and pH level of the aquatic environment 6.5 ± 0.1. The observations were conducted every hour on the first day and every 12 h on the second day.

### 3.6. Statistics

The statistical analysis is performed by OriginPro 8 and Microsoft Office Excel.

## 4. Conclusions

We have successfully prepared a series of zeolite materials with two different topologies, namely FAU and MFI, containing different amounts of silver, spanning between a few % by wt and up to 35%wt, in either ionic or metallic nanoparticle forms. The observed effects could be explained by the release of Ag species as ions and nanoparticles, and also, the ions originating from them were as follows:

The material with the highest silver content of mostly ionic silver species, the AgXas material, presented a strong bactericidal activity, showing a bactericidal effect at a concentration of 0.15 g/L^−1^ for *E. coli* *ATCC 25922* and *S. aureus* *ATCC 25923*. Its measured MIC is 0.003 g/L^−1^ (99.999% growth inhibition) for both tested strains. At an even lower concentration of 0.0015 g/L^−1^, it has an inhibitory effect on both bacteria too. SA25-Ag fresh and old dispersions, AgXcl fresh and old dispersions, and the AgXrc fresh dispersion had a bactericidal effect at a concentration of 2.5 g/L^−1^, indicating that the high silica ZSM-5 zeolite performs with a similar efficiency at an order of magnitude-lower silver content compared to the Faujasites loaded with silver nanoparticles. An aging effect of the prepared silver zeolites test dispersions was observed for the first time by us. For instance, AgXrc old, with a high silver content (365 wt), lost its antibacterial properties after one month of storage at 4 °C. *E. coli* *ATCC 25922* was significantly more sensitive than *S. aureus* *ATCC 25923*, according to the microbiological results obtained.

AgXas and AgXrc presented a good prooxidant activity according to the control of pure Ag nanoparticles (30–50 nm) and appear to be the best candidates and rivals of Ag nanoparticles in ROS generation or oxidizing reactions, especially in physiological conditions (pH 7.4/37 °C). This result explains their bactericidal effects.

On the contrary, all the tested materials, including AgXas and AgXrc in some cases and conditions, exhibited strong inhibitory effects when tested in the selected ROS-generating systems. These results support their application for the prevention of free radical oxidation.

The demonstrated effects suggest the possible biological applications of the newly synthesized silver-loaded zeolitic materials, releasing Ag species. Their bactericidal or bacteriostatic effects would be gentle in comparison to silver ions originating from pure nanoparticles, as mentioned by Chen (2019) [19]. Most of these materials would not advance the accumulation of ROS and would not burden the metabolic processes connected with their generation, breaking the extremely sensitive oxidation equilibrium.

*Daphnia magna* was proved to be a very sensitive biomarker of ecotoxicity.

## Figures and Tables

**Table 1:** Materials’ description, including sample batch notation, the parent zeolite Si-to-Al ratio, silver content, and the observed silver forms: isolated ionic species and metallic silver nanoparticles. The ivory color indicated for the zeolite–X materials comes from the binder material and is not specific to silver-exchanged X-zeolites, which are white.

Sample Material	Si/Al	Ag Cont. (EDX) %wt	Ag Form	Zeolite Type	Framework Type
AgXas	1.23	35	Ag^+^, some Ag^o^ (particles), ivory	X	FAU
AgXcl	1.23	35	mostly metallic Ag nanoparticles, black, some larger Ag metal clusters	X	FAU
AgXrc	1.23	35	Ag^+^, some metallic nanoparticles, a few larger Ag particles	X	FAU
SA12-Ag	12	3.5	Ag^+^, some Ag^o^ (particles), white	ZSM-5	MFI
SA25-Ag	25	2.5	Ag^+^, a few Ag^o^ (particles), white	ZSM-5	MFI
SA25-Ag_0.33_	25	1.2	Ag^+^, white	ZSM-5	MFI

**Table 2:** Results for *E. coli* *ATCC 25922* at the 4th h of impact with other zeolite materials, calculated in CFU/mL.

	Concentration	2.5 g/L^−1^	0.25 mg/L^−1^	0.025 g/L^−1^	0.0025 g/L^−1^
Material					
SA12-Ag fresh		0	>4 × 10^7^	2 × 10^8^	1 × 10^8^
SA12-Ag old		0	>4 × 10^7^	2 × 10^8^	2 × 10^8^
AgXcl fresh		0	>4 × 10^4^	2 × 10^8^	2 × 10^8^
AgXcl old		0	>4 × 10^4^	2 × 10^8^	2 × 10^8^
AgXrc fresh		0	2 × 10^8^	2 × 10^8^	2 × 10^8^
AgXrc old		>4 × 10^4^	2 × 10^8^	2 × 10^8^	2 × 10^8^
control		1.14 × 10^8^	1.14 × 10^8^	1.14 × 10^8^	1.14 × 10^8^

**Table 3:** Results for *E. coli* *ATCC 25922* at the 24th h of impact with zeolites AgXas, SA25-Ag, and SA Ag_0.33_, calculated in CFU/mL.

	Concentration	0.15 g/L^−1^	0.003 g/L^−1^	0.0015 g/L^−1^	0.0007 g/L^−1^
Material					
AgXas		0	3.85 × 10^8^	>4 × 10^9^	>4 × 10^9^
SA25-Ag		1 × 10^1^	>4 × 10^8^	>4 × 10^9^	1.15 × 10^9^
SA25-Ag_0.33_		>4 × 10^4^	>1 × 10^9^	>1 × 10^9^	1.15 × 10^9^
control		1.11 × 10^9^	1.11 × 10^9^	1.11 × 10^9^	1.11 × 10^9^

**Table 4:** Results for *E. coli* *ATCC 25922* at the 24th h of impact with other zeolite materials, calculated in CFU/mL.

	Concentration	2.5 g/L^−1^	0.25 g/L^−1^	0.025 g/L^−1^	0.0025 g/L^−1^
Material					
SA12-Ag fresh		0	0	0	1.9 × 10^9^
SA-12Ag old		0	>4 × 10^7^	2 × 10^8^	3.2 × 10^8^
AgXcl fresh		0	0	0	1 × 10^7^
AgXcl old		0	0	0	2 × 10^7^
AgXrc fresh		0	0	2 × 10^8^	2 × 10^8^
AgXrc old		>4 × 10^4^	2 × 10^8^	2 × 10^8^	4 × 10^9^
control		1.41 × 10^9^	1.41 × 10^9^	1.41 × 10^9^	1.41 × 10^9^

**Table 5:** Results for *S. aureus* *ATCC 25923*, at the 4th h of impact, calculated in CFU/mL.

	Concentration	0.15 g/L^−1^	0.003 g/L^−1^	0.0015 g/L^−1^	0.007 g/L^−1^
Material					
AgXas		0	4 × 10^4^	>4 × 10^8^	>4 × 10^8^
SA25-Ag		>4 × 10^4^	>4 × 10^8^	>4 × 10^8^	>5 × 10^8^
SA25-Ag_0.33_		2 × 10^5^	4 × 10^8^	>5 × 10^8^	>5 × 10^8^
control		8.2 × 10^5^	8.2 × 10^5^	8.2 × 10^5^	8.2 × 10^5^

**Table 6:** Results for *S. aureus* 25923, at the 4th h of impact, calculated in CFU/mL.

	Concentration	2.5 g/L^−1^	0.25 g/L^−1^	0.025 g/L^−1^	0.0025 g/L^−1^
Material					
SA12-Ag fresh		0	1.3 × 10^7^	1 × 10^8^	2 × 10^8^
SA12-Ag old		0	2 × 10^7^	2 × 10^8^	2 × 10^8^
AgXcl fresh		0	0	1 × 10^7^	2 × 10^8^
AgXcl old		0	1 × 10^7^	2 × 10^8^	2 × 10^9^
AgXrc fresh		0	2 × 10^8^	2 × 10^8^	2 × 10^9^
AgXrc old		>4 × 10^4^	2 × 10^8^	2 × 10^9^	2 × 10^9^
control		1.21 × 10^9^	1.21 × 10^9^	1.21 × 10^9^	1.21 × 10^9^

## Data Availability

Data are contained within the article.

## Supplementary Materials

The following supporting information can be downloaded at: https://www.mdpi.com/article/10.3390/ph17121586/s1, Figure S1. Powder diffraction pattern of the studied Ag-X materials compared to computed patterns for the FAU (Na-X) parent zeolite structure, FCC Ag-metal, hexagonal Ag-metal phase, and Ag2O; Figure S2. Same as on Figure S2, but around the high angle part of the pattern; Figure S3. PXRD of the lowest level silver loading ZSM-5 material with Si/Al=25, compared to the computed pattern of FCC metallic silver; Figure S4. PXRD patterns of all studied silver exchanged ZSM-5 zeolites, along with the computed pattern of the MFI prototype; Figure S5. Same as on Figure S4 but focused on the higher angles in the pattern, shown along with the computed pattern of the FCC phase metallic silver. As seen in both Figures S4 and S5, no extra phases are observed in the ZSM-5 Ag-exchanged materials.
