# Supplementary material for: Antimicrobial and Oxidative Activities of Different Levels of Silver-Exchanged Zeolites X and ZSM-5 and Their Ecotoxicity
Source: Pharmaceuticals (Basel). 2024 Nov 25;17(12):1586. doi: 10.3390/ph17121586 (PMC11676760; doi:10.3390/ph17121586)
Supplement: Supplementary file 1 [file pharmaceuticals-17-01586-s001.zip › pharmaceuticals-3278954-supplementary.pdf]

**Supplementary material for:**

# Antimicrobial and Oxidative Activities of Different Level Silver-Exchanged Zeolites X and ZSM-5 and Their Ecotoxicity

Elitsa L. Pavlova[1], Elena P. Nenova[2], Lyubomira D. Yocheva[3], Iliana A. Ivanova[2], Peter A. Georgiev[1]

[1]*Faculty of Physics, Sofia University"St. Kliment Ohridski", 5 James Bourchier Blvd., 1164 Sofia, Bulgaria*

[2]*Faculty of Biology, Sofia University "St. Kliment Ohridski", 8 Dragan Tsankov Blvd, 1164 Sofia, Bulgaria*

[3]*Faculty of Medicine, Sofia University "St. Kliment Ohridski, 1 Kozyak Str, 1407, Sofia, Bulgaria*

* Corresponding authors:
Elitsa Pavlova, elli_pavlova@abv.bg, +359 887 982 172
Peter Georgiev, pageorgiev@phys.uni-sofia.bg

**Powder X-Ray Diffraction (PXRD) patterns of the investigated materials**

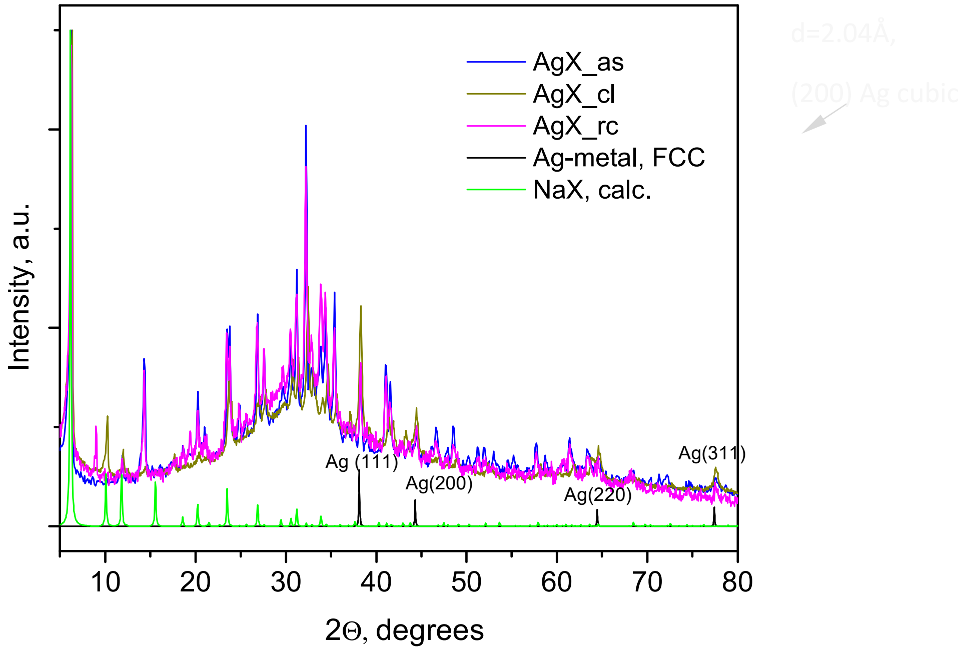


**Figure S1**. Powder diffraction pattern of the studied Ag-X materials compared to computed patterns for the FAU (Na-X) parent zeolite structure, FCC Ag-metal, hexagonal Ag-metal phase, and $Ag_2O$.

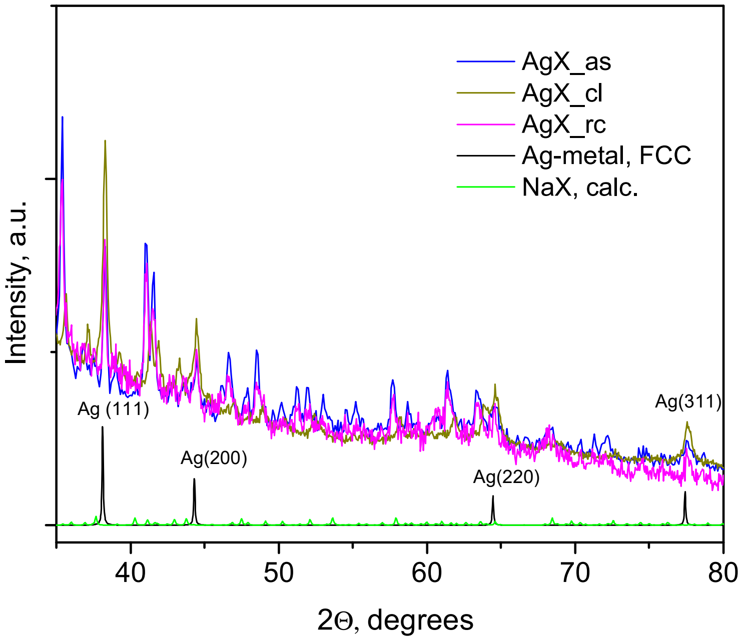


**Figure S2.** Same as on Figure S2, but around the high angle part of the pattern.

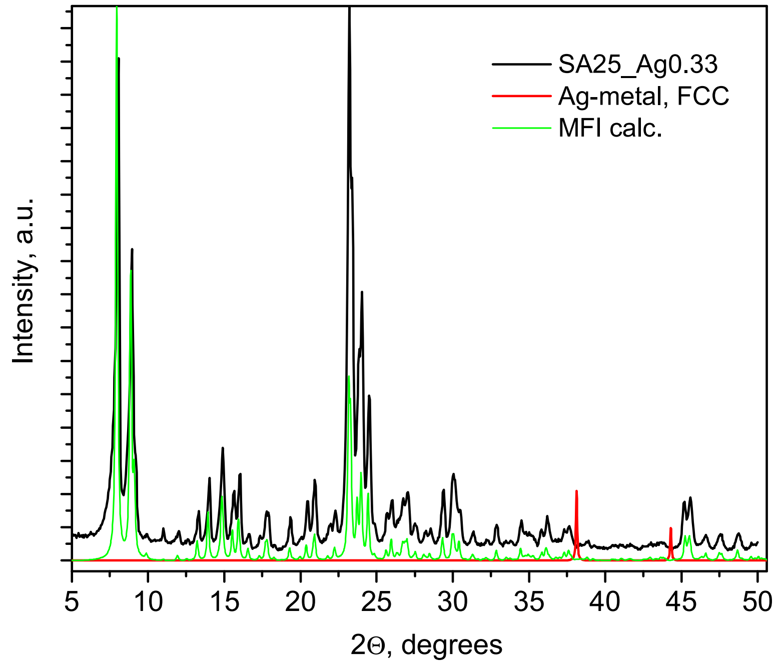


**Figure S3.** PXRD of the lowest level silver loading ZSM-5 material with Si/Al=25, compared to the computed pattern of FCC metallic silver.

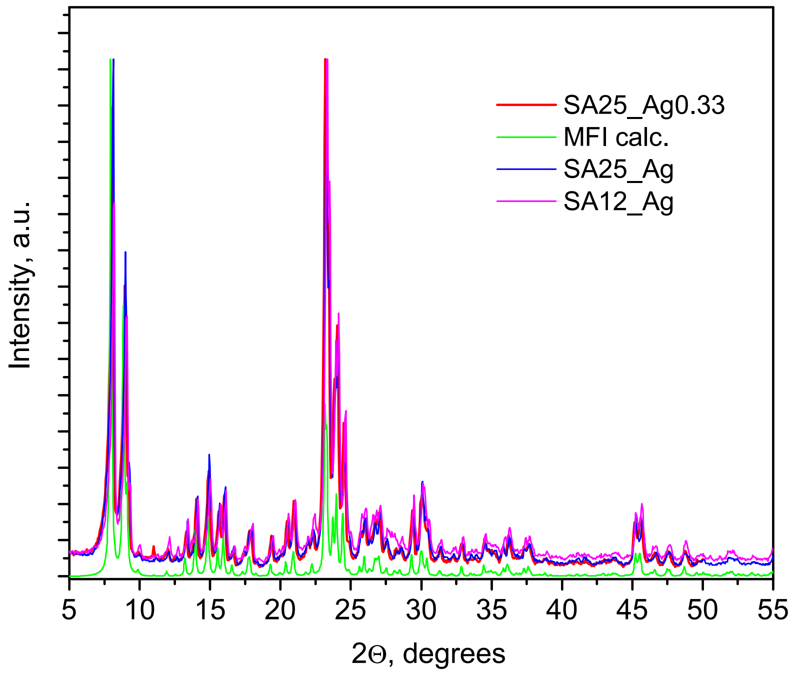


**Figure S4.** PXRD patterns of all studied silver exchanged ZSM-5 zeolites, along with the computed pattern of the MFI prototype.

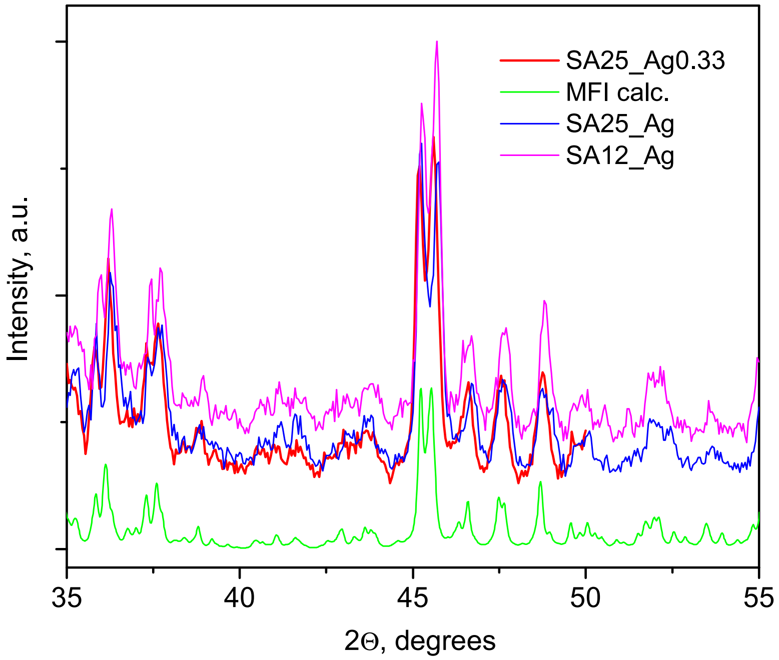


**Figure S5.** Same as on Figure S4 but focused on the higher angles in the pattern, shown along with the computed pattern of the FCC phase metallic silver. As seen in both Figures S4 and S5, no extra phases are observed in the ZSM-5 Ag-exchanged materials.
